# Role of cannabinoids in the regulation of bone remodeling

**DOI:** 10.3389/fendo.2012.00136

**Published:** 2012-11-16

**Authors:** Aymen I. Idris, Stuart H. Ralston

**Affiliations:** ^1^Bone and Cancer Group, Edinburgh Cancer Research Centre, The University of EdinburghEdinburgh, UK; ^2^Rheumatic Disease Unit, The Centre for Molecular Medicine, The University of EdinburghEdinburgh, UK

**Keywords:** cannabinoid, Bone, CB_1_, CB_2_, GPR55, Osteoblasts, Osteoclasts, Adipocytes

## Abstract

The endocannabinoid system plays a key role in regulating a variety of physiological processes such as appetite control and energy balance, pain perception, and immune responses. Recent studies have implicated the endocannabinoid system in the regulation of bone cell activity and bone remodeling. These studies showed that endogenous cannabinoid ligands, cannabinoid receptors, and the enzymes responsible for ligand synthesis and breakdown all play important roles in bone mass and in the regulation of bone disease. These findings suggest that the endocannabinoid pathway could be of value as a therapeutic target for the prevention and treatment of bone diseases. Here, we review the role of the skeletal endocannabinoid system in the regulation of bone remodeling in health and disease.

## INTRODUCTION

The endogenous cannabinoid (endocannabinoid) system is a complex network of receptors, a variety of ligands, and a series of enzymes that are responsible for ligand synthesis and breakdown. Endocannabinoids and their receptors are involved in the regulation of numerous physiological processes including neurotransmission, pain perception, learning, memory, cardiovascular homeostasis, appetite, motor function, and the immune response (reviewed in Klein et al., 2000; Grant and Cahn, 2005; Di Marzo, 2008). There is accumulating evidence to suggest that endocannabinoids and their receptors play important roles in bone metabolism by regulating bone mass, bone loss, and bone cell function. This review summarizes *in vitro* and *in vivo* findings relating to the action of cannabinoid ligands in the skeleton.

## THE SKELETAL ENDOCANNABINOID SYSTEM

### CANNABINOID RECEPTOR LIGANDS IN BONE

Cannabinoid receptor ligands can be classified into three groups based on their source of production; endogenous cannabinoids (endocannabinoids), phytocannabinoids, and synthetic cannabinoids. Two of the best characterized endocannabinoids are *N*-arachidonoylethanolamine (anandamide) and 2-arachidonoylglycerol (2-AG). The endocannabinoids anandamide and 2-AG are responsible for most pharmacological actions associated with cannabinoid receptors in mammalian cells (Reviewed in Pertwee, 2005). Anandamide and 2-AG are both derivatives of arachidonic acid and are produced from breakdown of glycerophospholipids in the cell membrane. Anandamide is synthesized from *N*-arachidonoyl phosphatidylethanolamine (NAPE) by the enzyme NAPE-phospholipase D (NAPE-PLD), whereas synthesis of 2-AG occurs through the action of phospholipase C (PLC) and the diacylglycerol lipases alpha and beta (DAGLα and DAGLβ) on membrane phospholipids (Di Marzo et al., 1996; Maejima et al., 2001a,b; Simon and Cravatt, 2006). Once formed, endocannabinoids are transported across cell membranes by passive diffusion or endocytosis (for extensive review, refer to Fowler, 2012; Hermann et al., 2006). Anandamide and 2-AG are highly expressed in the brain and are also detected in a number of peripheral tissues including heart, liver, kidney, testis, and blood (Felder et al., 1993, 1996; Stella et al., 1997; Kondo et al., 1998; Di Marzo et al., 2002; Ross, 2003; van der Stelt and Di Marzo, 2005; Tam et al., 2008). Endocannabinoids have a short half life due to the fact that they are rapidly degraded by a variety of enzymes including fatty acid amide hydrolase (FAAH) and monoacylglycerol lipase (MGAL; Dinh et al., 2002). It should be noted, however, that FAAH is not specific for cannabinoids and degrades many other lipid containing molecules (Basavarajappa, 2007).

There is evidence that the endocannabinoids 2-AG and anandamide are produced endogenously in the bone marrow and within the metabolically active trabecular compartment (Bab et al., 2008; Tam et al., 2008). A number of studies have shown that osteoblasts and osteoclasts are capable of producing anandamide and 2-AG in culture. Ridge et al. (2007) reported in abstract form that cultured osteoblast-like cells MC3T3-E1 and mouse osteoclasts produced 2-AG *in vitro* and that anandamide was produced by cultured osteoblasts but not osteoclasts. A recent study by the same group reported that the differentiation of human osteoclasts from monocytes is associated with a reduction in 2-AG levels and an increase in anandamide levels (Whyte et al., 2012). Rossi et al. (2009) reported that cultured human osteoclasts produced 2-AG and detectable quantities of anandamide; levels of both cannabinoids increased when the cultures were treated with the FAAH inhibitor URB597. Taken together, these observations indicate that 2-AG and anandamide are probably produced locally within bone and by bone cells in culture. In a study by Richardson et al. (2008), neither 2-AG nor anandamide were detected in synovial fluid from normal subjects, but both endocannabinoids were detected in synovial fluid from patients with osteoarthritis (OA) and rheumatoid arthritis (RA). Interestingly 2-AG levels in this study were higher in patients with OA as compared with RA.

Cannabinoid receptors are also activated by plant derived cannabinoids termed phytocannabinoids. The *Cannabis sativa* plant contains a large number of phytocannabinoids such as Δ^9^-tetrahydrocannabinol (THC) which is an agonist at CB_1_ and CB_2_ receptors. It should be noted, however, that many phytocannabinoids such as cannabidiol bind weakly to cannabinoid receptors (reviewed by Mechoulam, 2005). A large number of synthetic cannabinoids have also been prepared some of which such as CP55,940, JWH133, and HU308 act as agonists thereby mimicking the action of endocannabinoids at a number of targets (Pertwee, 2005). On the other hand, a variety of synthetic compounds including SR141716A (also known as Rimonabant), AM251, and AM630 are described as inverse agonists/antagonists due to their ability to down-regulate the activity of cannabinoid receptors in the presence and absence of agonist binding (Gatley et al., 1996, 1997; Bouaboula et al., 1997; Hosohata et al., 1997a,b; Landsman et al., 1998; Lan et al., 1999; Ross et al., 1999; Meschler et al., 2000). For a comprehensive list of pharmacological properties of some of the most important cannabinoid receptor ligands refer to (Pertwee, 2005, 2010).

### CANNABINOID RECEPTORS IN BONE

Endocannabinoids and their synthetic analogs bind to and activate two known cannabinoid receptors: CB_1_ and CB_2_ (Maccarrone and Finazzi-Agro, 2002; Pertwee and Ross, 2002). Recent studies suggest that the “orphan” G protein-coupled receptor GPR55 might represent a third cannabinoid receptor (Begg et al., 2005; Ryberg et al., 2007). The CB_1_ receptor, encoded by the *CNR1* gene was the first cannabinoid receptor to be identified and it’s mainly expressed in the brain (Matsuda et al., 1990). In the skeleton, CB_1_ receptors are expressed on nerve fibers intervening bone (Tam et al., 2006, 2008) and on cells of the immune system within the BM compartment (Klein et al., 2000, 2003). We and others reported that CB_1_ receptors are also present on osteoblasts, osteoclasts, and BM derived adipocytes at both protein and mRNA levels (Idris et al., 2009; Rossi et al., 2009). The CB_2_ receptor encoded by the *CNR2* gene was originally identified in macrophages in the marginal zone of the spleen (Munro et al., 1993) but is now known to be expressed in many other tissues including bone and synovial joints as well as some regions of the central nervous system (Bouaboula et al., 1993; Galiegue et al., 1995; Pertwee, 1997; Nong et al., 2001; Klein et al., 2003; Idris et al., 2005; Ofek et al., 2006; Scutt and Williamson, 2007; Palazuelos et al., 2008). CB_2_ receptors are also expressed by osteoblasts, osteoclasts, and osteocytes at significantly higher level than that reported for CB_1_ (Idris et al., 2005; Ofek et al., 2006; Rossi et al., 2009; Whyte et al., 2012). Recent studies reported that bone cells including osteoblasts and osteoclasts also express GPR55 which is known to be targeted by endocannabinoids and synthetic cannabinoid ligands (Smart et al., 2000; Saunders et al., 2007; Abed et al., 2009; Rossi et al., 2009; Whyte et al., 2009). The GPR55 receptor encoded by the *GRP55* gene is widely expressed but the highest levels are detected in the adrenals, the gastrointestinal tract, and the brain (Ryberg et al., 2007). It should be noted that cannabinoids such as anandamide can bind to other receptors such as nicotinic acetylcholine receptors, calcium channels, voltage-gated potassium channels, and transient receptor potential vanilloid receptors (TRPVs; Di Marzo et al., 2002; van der et al., 2005), although the physiological significance of this in the skeleton is unclear.

### CANNABINOID RECEPTOR SIGNALING IN BONE

Cannabinoid receptors are a class of cell membrane receptors that belongs to the G protein-coupled receptor superfamily (Bouaboula et al., 1996; Ross et al., 1999). The CB_1_ receptor is constitutively active and therefore is able to transduce a biological signal in the absence of ligand (Carayon et al., 1998). Accordingly, cannabinoid receptor agonists inhibit adenylyl cyclase causing reduction in intracellular levels of cyclic adenosine monophosphate (cAMP; Demuth and Molleman, 2006). CB_1_ and CB_2_ receptors are also linked to a variety of other second messengers including nuclear factor of kappa B (NFκB), p38 mitogen-activated protein kinase (MAPK), extracellular signal-regulated kinases (ERKs), c-Jun N-terminal kinases (JNKs), PI3/Akt and focal adhesion kinase (FAK) phosphorylation (Derkinderen et al., 1996; Daaka et al., 1997; Guzman et al., 2001b; Ho et al., 2002; Molina-Holgado et al., 2002; Sanchez et al., 2003; Karanian et al., 2005; Demuth and Molleman, 2006). The CB_1_ receptor has also been shown to couple to generation of the lipid second messenger ceramide by sphingomyelin hydrolysis and through *de novo* synthesis of ceramide (Guzman et al., 2001a). Cannabinoid receptor antagonists/inverse agonists such as the CB_1_ selective AM251 and CB_2_ selective AM630 block the effects of cannabinoid receptor agonists as well as down-regulate the constitutive activity of cannabinoid receptors in the absence of agonist binding (Bouaboula et al., 1997, 1999). Very little is known about the signaling mechanisms used by cannabinoid receptors to influence bone cell activity. There is evidence that the CB_1_ receptor regulates osteoblast and adipocyte differentiation by modulating intracellular cAMP levels (Idris et al., 2009). Moreover, recent studies showed that CB_2_ selective agonists induce mitogenic effects in osteoblasts via activation of a Gi protein-cyclin D1 and ERK1/2 axis (Ofek et al., 2011; Sophocleous et al., 2011). However, the mechanisms by which the CB_1_ and CB_2_ receptor regulate osteoclast differentiation and activity have not yet been fully clarified.

The GPR55 receptor is coupled to the G_12_ family of proteins (Gα_12_ and Gα_13_) rather than the G_i/o_ proteins. The signaling pathways downstream of GPR55 have been less widely studied than CB_1_ and CB_2_ but ligand-induced activation of GPR55 has been shown to activate the small GTP binding proteins RhoA, Cdc42, and Rac1 (Ryberg et al., 2007), to trigger activation of the ERK/MAPK signaling cascade (Kapur et al., 2009); to elicit release of intracellular calcium through activation of PLC (Lauckner et al., 2008; Kapur et al., 2009) and to activate NFAT through effects on intracellular calcium (Ross, 2009). Activation of GPR55 with O-1602 and lysophosphatidylinositol (LPI) in osteoclasts has been reported to increase levels of active GTP-bound Rho and to stimulate ERK phosphorylation (Whyte et al., 2009).

## REGULATION OF OSTEOCLASTIC BONE RESORPTION BY CANNABINOIDS

The *classical* cannabinoid receptors CB_1_ and CB_2_ and the *orphan* receptor GPR55 all play significant roles in the regulation of osteoclast function and bone resorption. Idris et al. (2005) were first to report that genetic inactivation of the CB_1_ receptor results in high peak bone mass in young mice. In this study, a detailed micro-computed tomography scanning of bone and histomorphometric analysis of bone formation and resorption revealed that mice deficient in CB_1_ receptor have less osteoclasts and reduced bone resorption (Idris et al., 2005). These findings have led to the realization that cannabinoid receptors play a significant role in the regulation of peak bone mass. In keeping with these observations, the CB_1_ selective antagonist/inverse agonists AM251 and SR141716A have been reported to cause osteoclast apoptosis and inhibit osteoclast formation *in vitro* and osteoclasts cultured from CB_1_ deficient mice were found to be resistant to the effects of AM251 indicating that the mechanism of osteoclast inhibition was mediated at least in part, by the CB_1_ receptor (Idris et al., 2005). Further studies have shown that the CB_1_ selective antagonist/inverse agonist AM251 (1–3mg/kg/day) prevents ovariectomy induce bone loss in wild type mice and that CB_1_ deficient mice are resistant to ovariectomy-induced bone loss, findings which support the view that activation of the CB_1_ receptor promotes osteoclast differentiation and bone resorption (Idris et al., 2005). There is evidence that the defect in osteoclast formation in CB_1_ deficient mice is caused both, by a reduction in the sensitivity of osteoclast precursors to RANKL and a reduction in the ability of CB_1_ deficient osteoblasts to support osteoclast formation due to reduced RANKL expression (Idris et al., 2009).

The CB_2_ receptor also regulates osteoclast activity and bone resorption, but research in this area to date has yielded rather contradictory results. Ofek et al. (2006) reported that CB_2_ deficient mice developed osteoporosis with increasing age due to increased bone turnover. In keeping with these findings it was reported in the same study that the CB_2_ selective agonist HU308 inhibited RANKL-induced osteoclast formation in bone marrow and RAW 264.7 cultures* in vitro*. In complete contrast to these findings, Idris and colleagues reported, that anandamide and 2-AG, HU308 and JWH133 enhanced M-CSF- and RANKL-induced osteoclast formation over the concentration range 1–1000 nM (Idris et al., 2005), whereas the CB_2_ selective antagonist/inverse agonist AM630 was inhibitory (Idris et al., 2005, 2008). Further studies by Idris et al. (2008) showed that bone marrow cells isolated from CB_2_ deficient mice produce fewer osteoclasts in response to RANKL than wild type controls and that CB_2_ deficient mice were partially protected from ovariectomy-induced bone loss as compared with wild type littermates. In agreement with these observations others have reported that the endocannabinoids 2-AG and anandamide were found to stimulate bone resorption by human osteoclasts *in vitro* (Ridge et al., 2007; Whyte et al., 2012). Schuehly et al. (2011) have recently introduced a new class of highly CB_2_ selective ligands that strongly inhibited RANKL stimulated osteoclastogenesis in murine and human cultures. In the same study, the authors went to demonstrate that endocannabinoids stimulate osteoclast formation and these effects were significantly inhibited by natural biphenyl neolignan derivatives (Schuehly et al., 2011). *In vivo*, Idris et al. (2008) showed that the CB_2_ selective antagonist/inverse agonist AM630 prevents from ovariectomy-induced bone loss in a CB_2_ dependent and independent manner depending on administered dose. In broad agreement with this, Geng et al. (2010) showed that AM630 protected against the development of titanium particle induced osteolytic bone loss by reducing osteoclastogenesis. Furthermore, Lunn et al. (2007) reported that the novel CB_2_ selective antagonist Sch.036 prevented bone damage in arthritic mice. Taken together these studies indicate that pharmacological inhibition of the CB_2_ receptor inhibits osteoclast formation and reduces bone loss in adult mice. However, Rossi et al. (2009) reported that the CB_2_ selective antagonist/inverse agonist AM630 at high concentrations (10 µM) stimulated human osteoclast formation. The stimulatory effects of AM630 on osteoclast formation in human cultures reported by Rossi and colleagues are exactly opposite to the inhibitory effects if AM630 on osteoclast formation in mouse cultures reported by Idris et al. (2005), 2008. The reasons for this are unclear but possibilities include; species differences in responsiveness to AM630, off-target effects of AM630 at the high concentrations that were used or factors such as the choice of serum, some of which contain bioactive amounts of the endocannabinoid 2-AG sufficient to influence osteoclast differentiation and activity (Marazzi et al., 2011).

Recent studies have shown that the GPR55 receptor regulates osteoclast activity and bone resorption. A study by Whyte et al. (2009) showed that the GPR55 agonists L-α-LPI and O-1602 both inhibited osteoclast formation from bone marrow macrophages *in vitro*, whereas the GPR55 antagonist cannabidiol increased osteoclast formation. Although GPR55 agonists were found in this study to inhibit osteoclast formation, they actually stimulated the resorptive activity of osteoclasts. Conversely, the GPR55 antagonist cannabidiol enhanced osteoclast formation and inhibited resorptive activity. In keeping with these observations male mice with targeted inactivation of GPR55 were found to have increased numbers of osteoclasts* in vivo*, but these appeared unable to resorb bone effectively since trabecular bone mass was increased and cartilage remnants at the growth plate were not resorbed efficiently. Rather surprisingly, female mice deficient in GPR55 were found to have reduced numbers of osteoclasts and increased amounts of unresorbed growth plate cartilage, but had normal bone mass (Whyte et al., 2009). Further studies in wild type mice revealed that cannabidiol reduced levels of CTX – a biochemical marker of bone resorption – consistent with the *in vitro* observations. Taken together these observations indicate that activation of GPR55 inhibits osteoclast formation, but increases the ability of osteoclasts to resorb bone. Conversely, inhibition of GPR55 appears to increase osteoclast formation but reduces the ability of osteoclasts to resorb bone. The cannabinoid derivative ajulemic acid (AJA) which is structurally similar to both CBD and THC has been found to inhibit formation of multinucleated TRAP positive cells in RANKL treated RAW264.7 cultures and in M-CSF and RANKL treated bone marrow macrophages (George et al., 2008). The mechanism by which AJA regulates osteoclast formation is unclear since it has weak affinity for CB_2_, and presumably does not activate CB_1_ in view of the fact that it lacks psychotropic properties. Possibilities would include an effect on PPARγ, which has recently been implicated in the regulation of osteoclast differentiation (Wan et al., 2007) or an effect on the GPR55 receptor.

In summary, there is evidence that both cannabinoid “*classical”* receptors CB_1_ and CB_2_ and the “*orphan”* receptor GPR55 regulate osteoclast activity and bone resorption. Conflicting results have been reported with regard to the role of CB_2_ in regulating osteoclast differentiation *in vitro* with stimulatory effects reported by some workers and inhibitory effects by others. The reasons for these differences remain unclear at present but one explanation may be the fact that many cannabinoid receptor ligands have complex pharmacology and are not entirely specific for the cannabinoid receptors they were originally designed to target. For example, AM251, which was classically considered to be specific CB_1_ selective antagonist/inverse agonist has recently been reported to act as a GPR55 agonist (Lauckner et al., 2008; Ross, 2009). Perplexingly SR141716A has been reported to act as a GPR55 antagonist (Kapur et al., 2009) or agonist (Lauckner et al., 2008), depending on the assay system used. Similarly, the synthetic cannabinoid agonist CP55,940 has been shown to be act as a GPR55 agonist/partial agonist and JWH015, formerly considered a specific CB_2_ agonist has been shown to be a GPR55 agonist (Kapur et al., 2009). These data indicate that the interpretation of some ligand activities previously attributed to effects on the CB_1_ or CB_2_ receptors may have been confounded by effects on the GPR55 receptor. Further research will clearly be required to investigate these issues and to explore the relative roles that CB_1_, CB_2_, and GPR55 play in regulating osteoclast differentiation and function.

## REGULATION OF OSTEOBLAST FUNCTION AND BONE FORMATION BY CANNABINOIDS

Endocannabinoids and their receptors are involved in the regulation of osteoblast differentiation and bone formation. Mice with targeted deletion of the CB_1_ receptor have been found to develop osteoporosis with increasing age due to reduced bone formation and accumulation of adipocytes in the bone marrow space (Idris et al., 2009). In these studies, bone marrow stromal cells from CB_1_ deficient mice had an increased capacity for adipocyte differentiation and a reduced capacity for osteoblast differentiation and these effects were reproduced by treatment of wild type cultures with the CB_1_ selective antagonist/inverse agonist AM251 (Idris et al., 2009). Furthermore, the CB_1_ selective antagonist/inverse agonist AM251 was found to block the stimulatory effect of CP55,490 on bone nodule formation *in vitro* (Idris et al., 2009). Signaling studies have shown that blockade of CB_1_ receptors with AM251 in osteoblast like cells and preadipocytes up-regulated cAMP, stimulated CREB phosphorylation, inhibited expression of the osteoblast-specific transcription factor RUNX2 and increased expression of the adipocyte-specific transcription factor PPARγ (Idris et al., 2009). Other research has shown that the CB_1_ receptor plays a key role in regulating the increased bone formation that accompanies traumatic brain injury (TBI). This was investigated by Tam et al. (2008) who reported that the expected increase in bone formation following TBI was absent in CB_1_ deficient mice, but was present in wild type and CB_2_ deficient mice. In this study, Tam et al. (2008) also reported that TBI induced bone formation in wild type mice was abolished by the beta adrenergic receptor agonist isoproterenol. This led the authors to speculate that CB_1_ receptors present on presynaptic nerve endings in bone might enhance bone formation by suppressing catecholamine release (Tam et al., 2008). Although this is a plausible hypothesis, the experiments conducted did not determine whether the effect of the CB_1_ receptor on bone formation was truly mediated by this sequence of events or not. Other studies by the same authors (Tam et al., 2006)**showed that bone formation rate and mineral apposition rate were reduced in young (9–12-week old) CB_1_ deficient mice on a CD1 background, again confirming that CB_1_ appears to play a key role in regulating bone formation. Recent findings have shown that the CB_1_ receptor plays a role in glucocorticoid-induced bone loss (Wu et al., 2011; Ko et al., 2012). CB_1_ blockage attenuated the deleterious actions of glucocorticoid treatment on osteoblast activity and bone formation, significantly reduced bone loss and abrogated marrow adiposity (Wu et al., 2011; Ko et al., 2012). Mechanistic studies revealed that CB_1_ regulates glucocorticoid-induced dysfunction in osteoblasts via activation of a number of pathways including PI3/Akt, MAPK and runt-related transcription factor 2 and peroxisome proliferator-activated receptor 2 (Wu et al., 2011; Ko et al., 2012).

The CB_2_ receptor also plays a role in regulating bone formation. The CB_2_ selective agonists HU308, JWH133, and JWH015 have all been shown to stimulate bone nodule formation in bone marrow stromal cell cultures *in vitro*, although similar effects have been observed with non-selective agonists including anandamide, 2-AG, CP55,940, and WIN 55,212 (Ofek et al., 2006; Scutt and Williamson, 2007; Idris et al., 2009). A specific role for CB_2_ receptors in mediating these effects is supported by the fact that bone marrow stromal cells from CB_2_ deficient mice have a reduced capacity to differentiate into bone nodules when compared with those of wild type littermates. Although mice with targeted inactivation of CB_2_ have increased bone turnover, there is a relative defect in bone formation as evidenced by the fact that CB_2_ deficient mice develop age-related osteoporosis (Ofek et al., 2006). This is consistent with a model whereby CB_2_ is required for maintenance of normal bone formation in high bone turnover states.

The role of the GPR55 receptor on bone formation has not been extensively studied, but Whyte et al. (2009) found no significant abnormalities in histomorphometric indices of bone formation in GPR55 knockout mice and also reported that the GPR55 agonist O-1602 had little effect on bone nodule formation in mouse calvarial osteoblast cultures. In view of this it seems unlikely that GPR55 plays a major role in regulating bone formation. Several phytocannabinoids including cannabidiol, cannabinol, cannabidivarin, THC, and tetrahydrocannabivarin (THCV) have been reported to stimulate bone nodule formation, collagen production, and alkaline phosphatase activity in cultures of bone marrow stromal cells (Scutt and Williamson, 2007). At the present time, however, it is unclear to what extent these compounds are acting through cannabinoid receptors or other molecular targets. For example the phytocannabinoid THCV which was found in the above study to promote bone nodule formation is known to act as an antagonist of CB_1_ and CB_2_ receptors, which would be expected to reduce bone formation, according to the studies that have been performed in genetically modified mice (Ofek et al., 2006; Tam et al., 2006; Idris et al., 2009). In view of this it is clear that further research will be required to fully investigate the mechanisms by which these phytocannabinoids regulate bone cell activity.

In summary, current evidence suggests that both the CB_1_ and CB_2_ receptors play significant roles in regulating osteoblast differentiation and bone formation in response to TBI and ageing. The fact that CB_1_ knockout mice develop accumulation of marrow fat also suggests that the CB_1_ receptor regulates the age-related “switch” in differentiation potential of bone marrow stromal cells (Gimble et al., 2006).

## REGULATION OF BONE MASS BY CANNABINOID RECEPTORS

A number of genetic and pharmacological studies have reported that cannabinoid receptors regulate bone mass in health and disease. Mice with deletion of the CB_1_, CB_2_, and GPR55 receptors all exhibit abnormalities of bone mass although this is dependent to an extent, on background strain, gender and age. The first report of an abnormality in bone mass in relation to the endocannabinoid system came from the studies of Idris and colleagues who reported that female CB_1_ deficient mice on inbred (ABH) and outbred (CD1) backgrounds exhibited high peak bone mass affecting the trabecular compartment of bone (Idris et al., 2005). This was found to be due to a defect in osteoclastic bone resorption and in keeping with this, CB_1_ deficient mice were found to be resistant to ovariectomy induced bone loss (Idris et al., 2005). In a subsequent study, Idris et al. (2009) demonstrated that the high peak bone mass in CB_1_ deficient mice was found in both genders, and went on to show that CB_1_ deficient mice developed marked trabecular osteoporosis with increasing age due to a defect in bone formation and accumulation of marrow fat. Remarkably, this age-related bone loss occurred despite the fact that osteoclastic bone resorption remained lower in CB_1_ deficient mice than in wild type littermates throughout life. In another study Tam et al. (2006) confirmed that CB_1_ deficient mice on a CD1 background had high peak bone mass. Although this was observed in both genders, the difference was significant only for male mice. In stark contrast to these findings, CB_1_ deficient mice on a C57BL/6 background were found by Tam et al. (2006) to have reduced peak bone mass when compared with wild type littermates. The molecular mechanisms for these differences remain to be fully explored but they are probably related to polymorphisms or mutations in genes that interact with cannabinoid receptors to regulate cellular responses in different mouse strains. Abnormalities of bone mass have also been described in CB_2_ deficient mice. The first report came from Ofek et al. (2006) who found no major abnormalities of bone mass in young (8-week old) mice, but found that by 51 weeks of age, the CB_2_ deficient mice had developed marked trabecular osteoporosis with cortical expansion. Histomorphometric examination showed evidence of high bone turnover indicating that the likely mechanisms of bone loss was relative uncoupling of bone resorption and bone formation (Ofek et al., 2006). Idris et al. (2008) also found that peak bone mass was relatively normal in CB_2_ deficient mice and recent studies have shown these mice develop age-related osteoporosis (Sophocleous et al., 2011).

In a recent study, Sophocleous et al. (2012) have reported in an abstract form that combined deficiency of the CB_1_ and CB_2_ receptors enhances peak bone mass but increases age-related bone loss. At 3 months of age, female CD1 mice deficient in both CB_1_ and CB_2_ receptors had significantly higher peak bone mass than wild type controls due to a significant decrease in osteoclast number and activity. Interestingly, these differences in peak bone mass and bone resorption observed were quantitatively similar to those previously observed in single knockouts of CB_1_ (Idris et al., 2005, 2009) and CB_2_ (Sophocleous et al., 2012) in the same background. By 12 months of age female deficient in both CB_1_ and CB_2_ receptors had significantly lower trabecular bone mass and histomorphometric analysis showed that this was associated with a dramatic increase in bone marrow fat accumulation and a reduction in osteoblast numbers and bone formation rate compared to wild type controls of similar age. The differences in bone mass and bone cell activity that the authors observed in female deficient in both CB_1_ and CB_2_ receptors were quantitatively similar to those previously observed in single knockouts of CB_1_ but not CB_2_ (Idris et al., 2009). Altogether, these data indicate that combined CB_1_ and CB_2_ deficiency enhances peak bone mass by an effect on bone resorption but predisposes to age-related osteoporosis by promoting adipocyte differentiation at the expense of osteoblast differentiation in the bone marrow compartment. This study demonstrates that CB_1_ and CB_2_ have overlapping but distinctive roles in skeletal homeostasis and show that CB_1_ in particular plays a key role in regulating osteoblast and adipocyte differentiation in the bone marrow compartment. Mice with targeted inactivation of the GPR55 receptor have been reported to have high peak bone mass affecting the trabecular compartment of the tibia and femur, but interestingly this was only noted in male mice (Whyte et al., 2009). The mechanism seemed to be impairment of bone resorption; although the reasons responsible for the gender difference in skeletal phenotype in these animals remains unclear at present.

## FUTURE RESEARCH DIRECTIONS

There is a steadily growing body of evidence suggesting that the skeletal endocannabinoid system plays a significant role in regulating bone mass and bone turnover. Several outstanding questions remain unanswered however. One is to define the mechanisms by which endocannabinoid production in bone is regulated and specifically to determine if it is influenced by classical calcium regulating hormones, cytokines and mechanical loading. Further research is also required to fully define the signaling pathways used by these receptors to regulate bone cell activity. There is evidence to suggest that the CB_1_ receptor regulates osteoblast and adipocyte differentiation through a cAMP-mediated pathway but little is known about the mechanisms by which cannabinoids regulate osteoclast activity. There have been major discrepancies between different studies with regard to the effects of different cannabinoid receptor ligands on osteoclast differentiation and function. These remain to be completely resolved but some of the discrepancies between studies could be due to the fact that many cannabinoid receptor ligands which were previously thought to be specific for CB_1_ and/or CB_2_ have now been shown to have effects on GPR55 signaling (Kapur et al., 2009; Pertwee, 2009; Ross, 2009). A further area of research which remains to be explored is to determine to what extent the CB_1_ receptor exerts its effects on the skeleton by a central (neuronal) or peripheral mechanism. This is clinically relevant since if the peripheral effects were predominant, it may be possible to develop agonists of CB_1_ that do not cross the blood brain barrier that could favorably influence bone formation without causing adverse psychotropic effects. The outcome of these studies will greatly enhance our understanding of the role of the skeletal endocannabinoid system in bone remodeling and encourage the development of new treatments for bone diseases based on targeting cannabinoid receptors.

## Conflict of Interest Statement

The authors are co-inventors on patents claiming the use of cannabinoid receptor ligands as treatments for bone disease.

## References

[B1] Abed E. Labelle D. Martineau C. Loghin A. Moreau R. (2009). Expression of transient receptor potential (TRP) channels in human and murine osteoblast-like cells. *Mol. Membr. Biol.* 26 146–158 19115145 10.1080/09687680802612721

[B2] Bab I. Ofek O. Tam J. Rehnelt J. Zimmer A. (2008). Endocannabinoids and the regulation of bone metabolism. *J. Neuroendocrinol.* 20(Suppl. 1) 69–74 18426503 10.1111/j.1365-2826.2008.01675.x

[B3] Basavarajappa B. S. (2007). Critical enzymes involved in endocannabinoid metabolism. *Protein Pept.Lett.* 14 237–246 17346227 10.2174/092986607780090829 PMC1939815

[B4] Begg M. Pacher P. Batkai S. Osei-Hyiaman D. Offertaler L. Mo F. M. (2005). Evidence for novel cannabinoid receptors. *Pharmacol.Ther.* 106 133–145 15866316 10.1016/j.pharmthera.2004.11.005

[B5] Bouaboula M. Dussossoy D. Casellas P. (1999). Regulation of peripheral cannabinoid receptor CB2 phosphorylation by the inverse agonist SR 144528. Implications for receptor biological responses. *J.Biol.Chem.* 274 20397–20405 10400664 10.1074/jbc.274.29.20397

[B6] Bouaboula M. Perrachon S. Milligan L. Canat X. Rinaldi-Carmona M. Portier M. (1997). A selective inverse agonist for central cannabinoid receptor inhibits mitogen-activated protein kinase activation stimulated by insulin or insulin-like growth factor 1. Evidence for a new model of receptor/ligand interactions. *J. Biol. Chem.* 272 22330–22339 9268384 10.1074/jbc.272.35.22330

[B7] Bouaboula M. Poinot-Chazel C. Marchand J. Canat X. Bourrie B. Rinaldi-Carmona M. (1996). Signaling pathway associated with stimulation of CB2 peripheral cannabinoid receptor. Involvement of both mitogen-activated protein kinase and induction of Krox-24 expression. *Eur. J. Biochem.* 237 704–711 8647116 10.1111/j.1432-1033.1996.0704p.x

[B8] Bouaboula M. Rinaldi M. Carayon P. Carillon C. Delpech B. Shire D. (1993). Cannabinoid-receptor expression in human leukocytes. *Eur.J.Biochem.* 214 173–180 8508790 10.1111/j.1432-1033.1993.tb17910.x

[B9] Carayon P. Marchand J. Dussossoy D. Derocq J. M. Jbilo O. Bord A. (1998). Modulation and functional involvement of CB2 peripheral cannabinoid receptors during B-cell differentiation. *Blood* 92 3605–3615 9808554

[B10] Chicca A. Marazzi J. Nicolussi S. Gertsch J. (2012). Evidence for bidirectional endocannabinoid transport across cell membranes. *J.Biol.Chem.* 287 34660–34682 22879589 10.1074/jbc.M112.373241 PMC3464571

[B11] Daaka Y. Zhu W. Friedman H. Klein T. W. (1997). Induction of interleukin-2 receptor alpha gene by delta9-tetrahydrocannabinol is mediated by nuclear factor kappaB and CB1 cannabinoid receptor. *DNA Cell Biol*. 16 301–309 9115639 10.1089/dna.1997.16.301

[B12] Demuth D. G. Molleman A. (2006). Cannabinoid signalling. *Life Sci.* 78 549–563 16109430 10.1016/j.lfs.2005.05.055

[B13] Derkinderen P. Toutant M. Burgaya F. Le B. M. Siciliano J. C. de F. (1996). Regulation of a neuronal form of focal adhesion kinase by anandamide. *Science* 273 1719–1722 8781236 10.1126/science.273.5282.1719

[B14] Di Marzo V. (2008). Targeting the endocannabinoid system: to enhance or reduce? *Nat. Rev. Drug Discov.* 7 438–455 18446159 10.1038/nrd2553

[B15] Di Marzo V. De Petrocellis L. Fezza F. Ligresti A. Bisogno T. (2002). Anandamide receptors. *Prostaglandins Leukot. Essent. Fatty Acids* 66 377–391 12052051 10.1054/plef.2001.0349

[B16] Di Marzo V. De Petrocellis L. Sepe N. Buono A. (1996). Biosynthesis of anandamide and related acylethanolamides in mouse J774 macrophages and N18 neuroblastoma cells. *Biochem. J.* 316 977–984 8670178 10.1042/bj3160977 PMC1217444

[B17] Dinh T. P. Freund T. F. Piomelli D. (2002). A role for monoglyceride lipase in 2-arachidonoylglycerol inactivation. *Chem. Phys. Lipids* 121 149–158 12505697 10.1016/s0009-3084(02)00150-0

[B18] Felder C. C. Briley E. M. Axelrod J. Simpson J. T. Mackie K. Devane W. A. (1993). Anandamide, an endogenous cannabimimetic eicosanoid, binds to the cloned human cannabinoid receptor and stimulates receptor-mediated signal transduction. *Proc. Natl. Acad. Sci. U.S.A.* 90 7656–7660 8395053 10.1073/pnas.90.16.7656 PMC47201

[B19] Felder C. C. Nielsen A. Briley E. M. Palkovits M. Priller J. Axelrod J. (1996). Isolation and measurement of the endogenous cannabinoid receptor agonist, anandamide, in brain and peripheral tissues of human and rat. *FEBS Lett.* 393 231–235 8814296 10.1016/0014-5793(96)00891-5

[B20] Fowler C. J. (2012). Anandamide uptake explained? *Trends Pharmacol.Sci.* 33 181–185 22297258 10.1016/j.tips.2012.01.001

[B21] Galiegue S. Mary S. Marchand J. Dussossoy D. Carriere D. Carayon P. (1995). Expression of central and peripheral cannabinoid receptors in human immune tissues and leukocyte subpopulations. *Eur.J.Biochem.* 232 54–61 7556170 10.1111/j.1432-1033.1995.tb20780.x

[B22] Gatley S. J. Gifford A. N. Volkow N. D. Lan R. Makriyannis A. (1996). 123I-labeled AM251: a radioiodinated ligand which binds in vivo to mouse brain cannabinoid CB1 receptors. *Eur. J. Pharmacol.* 307 331–338 8836622 10.1016/0014-2999(96)00279-8

[B23] Gatley S. J. Lan R. Pyatt B. Gifford A. N. Volkow N. D. Makriyannis A. (1997). Binding of the non-classical cannabinoid CP 55,940, and the diarylpyrazole AM251 to rodent brain cannabinoid receptors. *Life Sci.* 61 PL191-PL197 10.1016/s0024-3205(97)00690-5 9335234

[B24] Geng D. Xu Y. Yang H. Wang J. Zhu X. Zhu G. (2010). Protection against titanium particle induced osteolysis by cannabinoid receptor 2 selective antagonist. *Biomaterials* 31 1996–2000 20004468 10.1016/j.biomaterials.2009.11.069

[B25] George K. L. Saltman L. H. Stein G. S. Lian J. B. Zurier R. B. (2008). Ajulemic acid, a nonpsychoactive cannabinoid acid, suppresses osteoclastogenesis in mononuclear precursor cells, and induces apoptosis in mature osteoclast-like cells. *J. Cell Physiol.* 214 714–720 17786950 10.1002/jcp.21263

[B26] Gimble J. M. Zvonic S. Floyd Z. E. Kassem M. Nuttall M. E. (2006). Playing with bone and fat. *J.Cell. Biochem.* 98 251–266 16479589 10.1002/jcb.20777

[B27] Grant I. Cahn B. R. (2005). Cannabis and endocannabinoid modulators: therapeutic promises and challenges. *Clin. Neurosci. Res.* 5 185–199 18806886 10.1016/j.cnr.2005.08.015 PMC2544377

[B28] Guzman M. Galve-Roperh I. Sanchez C. (2001a). Ceramide: a new second messenger of cannabinoid action. *Trends Pharmacol. Sci.* 22 19–22 11165667 10.1016/s0165-6147(00)01586-8

[B29] Guzman M. Sanchez C. Galve-Roperh I. (2001b). Control of the cell survival/death decision by cannabinoids. *J. Mol. Med.* 78 613–625 11269508 10.1007/s001090000177

[B30] Hermann A. Kaczocha M. Deutsch D. G. (2006). 2-Arachidonoylglycerol (2-AG) membrane transport: history and outlook. *AAPS J.* 8 E409–E412 16808043 10.1007/BF02854913 PMC3231572

[B31] Ho B. Y. Current L. Drewett J. G. (2002). Role of intracellular loops of cannabinoid CB(1) receptor in functional interaction with G(alpha16). *FEBS Lett.* 522 130–134 12095632 10.1016/s0014-5793(02)02917-4

[B32] Hosohata K. Quock R. M. Hosohata Y. Burkey T. H. Makriyannis A. Consroe P. (1997a). AM630 is a competitive cannabinoid receptor antagonist in the guinea pig brain. *Life Sci.* 61 L115–L118 10.1016/s0024-3205(97)00596-1 9284087

[B33] Hosohata Y. Quock R. M. Hosohata K. Makriyannis A. Consroe P. Roeske W. R. (1997b). AM630 antagonism of cannabinoid-stimulated [35S]GTP gamma S binding in the mouse brain. *Eur. J. Pharmacol.* 321 R1–R3 9083796 10.1016/s0014-2999(97)00047-2

[B34] Idris A. I. Sophocleous A. Landao-Bassonga E. Canals M. Milligan G. Baker D. (2009). Cannabinoid receptor type 1 protects against age-related osteoporosis by regulating osteoblast and adipocyte differentiation in marrow stromal cells. *Cell Metab.* 10 139–147 19656492 10.1016/j.cmet.2009.07.006

[B35] Idris A. I. Sophocleous A. Landao-Bassonga E. van’t Hof R. J. Ralston S. H. (2008). Regulation of bone mass, osteoclast function and ovariectomy-induced bone loss by the type 2 cannabinoid receptor. *Endocrinology* 149 5619–5626 18635663 10.1210/en.2008-0150

[B36] Idris A. I. Van ’t Hof R. J. Greig I. R. Ridge S. A. Baker D. Ross R. A. (2005). Regulation of bone mass, bone loss and osteoclast activity by cannabinoid receptors. *Nat. Med.* 11 774–779 15908955 10.1038/nm1255 PMC1430341

[B37] Kapur A. Zhao P. Sharir H. Bai Y. Caron M. G. Barak L. S. (2009). Atypical responsiveness of the orphan receptor GPR55 to cannabinoid ligands. *J. Biol. Chem.* 284 29817–29827 19723626 10.1074/jbc.M109.050187 PMC2785612

[B38] Karanian D. A. Brown Q. B. Makriyannis A. Bahr B. A. (2005). Blocking cannabinoid activation of FAK and ERK1/2 compromises synaptic integrity in hippocampus. *Eur. J. Pharmacol.* 508 47–56 15680253 10.1016/j.ejphar.2004.12.009

[B39] Klein T. W. Lane B. Newton C. A. Friedman H. (2000). The cannabinoid system and cytokine network. *Proc. Soc. Exp. Biol. Med.* 225 1–8 10998193 10.1177/153537020022500101

[B40] Klein T. W. Newton C. Larsen K. Lu L. Perkins I. Nong L. (2003). The cannabinoid system and immune modulation. *J. Leukoc. Biol.* 74 486–496 12960289 10.1189/jlb.0303101

[B41] Ko J. Y. Wu R. W. Kuo S. J. Chen M. W. Yeh D. W. Ke H. C. (2012). Cannabinoid receptor 1 mediates glucocorticoid-induced bone loss in rats by perturbing bone mineral acquisition and marrow adipogenesis. *Arthritis Rheum.* 64 1204–1214 22127784 10.1002/art.33457

[B42] Kondo S. Kondo H. Nakane S. Kodaka T. Tokumura A. Waku K. (1998). 2-Arachidonoylglycerol, an endogenous cannabinoid receptor agonist: identification as one of the major species of monoacylglycerols in various rat tissues, and evidence for its generation through CA2+-dependent and -independent mechanisms. *FEBS Lett.* 429 152–156 9650580 10.1016/s0014-5793(98)00581-x

[B43] Lan R. Liu Q. Fan P. Lin S. Fernando S. R. McCallion D. (1999). Structure-activity relationships of pyrazole derivatives as cannabinoid receptor antagonists. *J. Med. Chem.* 42 769–776 10052983 10.1021/jm980363y

[B44] Landsman R. S. Makriyannis A. Deng H. Consroe P. Roeske W. R. Yamamura H. I. (1998). AM630 is an inverse agonist at the human cannabinoid CB1 receptor. *Life Sci.* 62 L109–L113 10.1016/s0024-3205(97)01187-9 9496703

[B45] Lauckner J. E. Jensen J. B. Chen H. Y. Lu H. C. Hille B. Mackie K. (2008). GPR55 is a cannabinoid receptor that increases intracellular calcium and inhibits M current. *Proc. Natl. Acad. Sci. U.S.A.* 105 2699–2704 18263732 10.1073/pnas.0711278105 PMC2268199

[B46] Lunn C. A. Fine J. Rojas-Triana A. Jackson J. V. Lavey B. Kozlowski J. A. (2007). Cannabinoid CB(2)-selective inverse agonist protects against antigen-induced bone loss. *Immunopharmacol. Immunotoxicol.* 29 387–401 18075852 10.1080/08923970701674997

[B47] Maccarrone M. Finazzi-Agro A. (2002). Endocannabinoids and their actions. *Vitam. Horm.* 65 225–255 12481549 10.1016/s0083-6729(02)65066-6

[B48] Maejima T. Hashimoto K. Yoshida T. Aiba A. Kano M. (2001a). Presynaptic inhibition caused by retrograde signal from metabotropic glutamate to cannabinoid receptors. *Neuron* 31 463–475 11516402 10.1016/s0896-6273(01)00375-0

[B49] Maejima T. Ohno-Shosaku T. Kano M. (2001b). Endogenous cannabinoid as a retrograde messenger from depolarized postsynaptic neurons to presynaptic terminals. *Neurosci.Res.* 40 205–210 11448511 10.1016/s0168-0102(01)00241-3

[B50] Marazzi J. Kleyer J. Paredes J. M. Gertsch J. (2011). Endocannabinoid content in fetal bovine sera – unexpected effects on mononuclear cells and osteoclastogenesis. *J. Immunol. Methods* 373 219–228 21920367 10.1016/j.jim.2011.08.021

[B51] Matsuda L. A. Lolait S. J. Brownstein M. J. Young A. C. Bonner T. I. (1990). Structure of a cannabinoid receptor and functional expression of the cloned cDNA. *Nature* 346 561–564 2165569 10.1038/346561a0

[B52] Mechoulam R. (2005). Plant cannabinoids: a neglected pharmacological treasure trove. *Br. J. Pharmacol.* 146 913–915 16205721 10.1038/sj.bjp.0706415 PMC1751232

[B53] Meschler J. P. Kraichely D. M. Wilken G. H. Howlett A. C. (2000). Inverse agonist properties of *N*-(piperidin-1-yl)-5-(4-chlorophenyl)-1-(2, 4-dichlorophenyl)-4-methyl-1H-pyrazole-3-carboxamide HCl (SR141716A) and 1-(2-chlorophenyl)-4-cyano-5-(4-methoxyphenyl)-1H-pyrazole-3-carboxyl ic acid phenylamide (CP-272871) for the CB(1) cannabinoid receptor. *Biochem. Pharmacol.* 60 1315–1323 11008125 10.1016/s0006-2952(00)00447-0

[B54] Molina-Holgado E. Vela J. M. revalo-Martin A. Almazan G. Molina-Holgado F. Borrell J. (2002). Cannabinoids promote oligodendrocyte progenitor survival: involvement of cannabinoid receptors and phosphatidylinositol-3 kinase/Akt signaling. *J. Neurosci.* 22 9742–9753 12427829 10.1523/JNEUROSCI.22-22-09742.2002 PMC6757831

[B55] Munro S. Thomas K. L. bu-Shaar M. (1993). Molecular characterization of a peripheral receptor for cannabinoids. *Nature* 365 61–65 7689702 10.1038/365061a0

[B56] Nong L. Newton C. Friedman H. Klein T. W. (2001). CB1 and CB2 receptor mRNA expression in human peripheral blood mononuclear cells (PBMC) from various donor types. *Adv. Exp. Med. Biol.* 206 229–233 11727770 10.1007/0-306-47611-8_27

[B57] Ofek O. Attar-Namdar M. Kram V. Dvir-Ginzberg M. Mechoulam R. Zimmer A. (2011). CB2 cannabinoid receptor targets mitogenic Gi protein-cyclin D1 axis in osteoblasts. *J. Bone Miner. Res.* 26 308–316 20803555 10.1002/jbmr.228 PMC3179350

[B58] Ofek O. Karsak M. Leclerc N. Fogel M. Frenkel B. Wright K. (2006). Peripheral cannabinoid receptor, CB2, regulates bone mass. *Proc. Natl. Acad. Sci. U.S.A.* 103 696–701 16407142 10.1073/pnas.0504187103 PMC1334629

[B59] Palazuelos J. Davoust N. Julien B. Hatterer E. Aguado T. Mechoulam R. (2008). The CB(2) cannabinoid receptor controls myeloid progenitor trafficking: involvement in the pathogenesis of an animal model of multiple sclerosis. *J. Biol. Chem.* 283 13320–13329 18334483 10.1074/jbc.M707960200

[B60] Pertwee R. G. (1997). Pharmacology of cannabinoid CB1 and CB2 receptors. *Pharmacol. Ther.* 74 129–180 9336020 10.1016/s0163-7258(97)82001-3

[B61] Pertwee R. G. (2005). Pharmacological actions of cannabinoids. *Handb. Exp. Pharmacol.* 1–51 16596770 10.1007/3-540-26573-2_1

[B62] Pertwee R. G. (2009). Emerging strategies for exploiting cannabinoid receptor agonists as medicines. *Br. J. Pharmacol.* 156 397–411 19226257 10.1111/j.1476-5381.2008.00048.x PMC2697681

[B63] Pertwee R. G. (2010). Receptors and channels targeted by synthetic cannabinoid receptor agonists and antagonists. *Curr. Med. Chem.* 17 1360–1381 20166927 10.2174/092986710790980050 PMC3013229

[B64] Pertwee R. G. Ross R. A. (2002). Cannabinoid receptors and their ligands. *Prostaglandins Leukot. Essent. Fatty Acids* 66 101–121 12052030 10.1054/plef.2001.0341

[B65] Richardson D. Pearson R. G. Kurian N. Latif M. L. Garle M. J. Barrett D. A. (2008). Characterisation of the cannabinoid receptor system in synovial tissue and fluid in patients with osteoarthritis and rheumatoid arthritis. *Arthritis Res. Ther.* 10 R43 10.1186/ar2401 PMC2453762 18416822

[B66] Ridge S. A. Ford L. Cameron G. A. Ross R. A. Rogers M. J. (2007). Endocannabinoids are produced by bone cells and stimulate bone resorption in vitro. *Bone* 40(Suppl. 1) S120

[B67] Ross R. A. (2003). Anandamide and vanilloid TRPV1 receptors. *Br. J. Pharmacol.* 140 790–801 14517174 10.1038/sj.bjp.0705467 PMC1574087

[B68] Ross R. A. (2009). The enigmatic pharmacology of GPR55. *Trends Pharmacol. Sci.* 30 156–163 19233486 10.1016/j.tips.2008.12.004

[B69] Ross R. A. Brockie H. C. Stevenson L. A. Murphy V. L. Templeton F. Makriyannis A. (1999). Agonist-inverse agonist characterization at CB1 and CB2 cannabinoid receptors of L759633, L759656, and AM630. *Br. J. Pharmacol.* 126 665–672 10188977 10.1038/sj.bjp.0702351 PMC1565857

[B70] Rossi F. Siniscalco D. Luongo L. De Petrocellis L. Bellini G. Petrosino S. (2009). The endovanilloid/endocannabinoid system in human osteoclasts: possible involvement in bone formation and resorption. *Bone* 44 476–484 19059369 10.1016/j.bone.2008.10.056

[B71] Ryberg E. Larsson N. Sjogren S. Hjorth S. Hermansson N. O. Leonova J. (2007). The orphan receptor GPR55 is a novel cannabinoid receptor. *Br. J. Pharmacol.* 152 1092–1101 17876302 10.1038/sj.bjp.0707460 PMC2095107

[B72] Sanchez M. G. Ruiz-Llorente L. Sanchez A. M. Diaz-Laviada I. (2003). Activation of phosphoinositide 3-kinase/PKB pathway by CB(1) and CB(2) cannabinoid receptors expressed in prostate PC-3 cells. Involvement in Raf-1 stimulation and NGF induction. *Cell Signal.* 15 851–859 12834810 10.1016/s0898-6568(03)00036-6

[B73] Saunders C. I. Kunde D. A. Crawford A. Geraghty D. P. (2007). Expression of transient receptor potential vanilloid 1 (TRPV1) and 2 (TRPV2) in human peripheral blood. *Mol.Immunol.* 44 1429–1435 16777226 10.1016/j.molimm.2006.04.027

[B74] Schuehly W. Paredes J. M. Kleyer J. Huefner A. Anavi-Goffer S. Raduner S. (2011). Mechanisms of osteoclastogenesis inhibition by a novel class of biphenyl-type cannabinoid CB(2) receptor inverse agonists. *Chem. Biol.* 18 1053–1064 21867920 10.1016/j.chembiol.2011.05.012

[B75] Scutt A. Williamson E. M. (2007). Cannabinoids stimulate fibroblastic colony formation by bone marrow cells indirectly via CB2 receptors. *Calcif. Tissue Int.* 80 50–59 17205329 10.1007/s00223-006-0171-7

[B76] Simon G. M. Cravatt B. F. (2006). Endocannabinoid biosynthesis proceeding through glycerophospho-*N*-acyl ethanolamine and a role for alpha/beta-hydrolase 4 in this pathway. *J. Biol. Chem.* 281 26465–26472 16818490 10.1074/jbc.M604660200

[B77] Smart D. Gunthorpe M. J. Jerman J. C. Nasir S. Gray J. Muir A. I. (2000). The endogenous lipid anandamide is a full agonist at the human vanilloid receptor (hVR1). *Br. J. Pharmacol.* 129 227–230 10694225 10.1038/sj.bjp.0703050 PMC1571834

[B78] Sophocleous A. Landao-Bassonga E. van’t Hof R. J. Idris A. I. Ralston S. H. (2011). The type 2 cannabinoid receptor regulates bone mass and ovariectomy-induced bone loss by affecting osteoblast differentiation and bone formation. *Endocrinology* 152 2141–2149 21447627 10.1210/en.2010-0930

[B79] Sophocleous A. Ralston S. H. Idris A. I. (2012). Combined deficiency of the CB1 and CB2 receptors enhances peak bone mass by inhibiting osteoclast differentiation but increases age-related bone loss by promoting adipocyte differentiation and reducing osteoblast differentiation. *Bone* 50 S32

[B80] Stella N. Schweitzer P. Piomelli D. (1997). A second endogenous cannabinoid that modulates long-term potentiation. *Nature* 388 773–778 9285589 10.1038/42015

[B81] Tam J. Ofek O. Fride E. Ledent C. Gabet Y. Muller R. (2006). Involvement of neuronal cannabinoid receptor, CB1, in regulation of bone mass and bone remodeling. *Mol. Pharmacol.* 70 786–792 16772520 10.1124/mol.106.026435

[B82] Tam J. Trembovler V. Di M. Petrosino V. S. Leo G. Alexandrovich A. (2008). The cannabinoid CB1 receptor regulates bone formation by modulating adrenergic signaling. *FASEB J.* 22 285–294 17704191 10.1096/fj.06-7957com

[B83] van der Stelt M Di Marzo V. (2005). Anandamide as an intracellular messenger regulating ion channel activity. *Prostaglandins Other Lipid Mediat.* 77 111–122 16099396 10.1016/j.prostaglandins.2004.09.007

[B84] van der S. M. Trevisani M. Vellani V. De P. L. Schiano M. A. Campi B. (2005). Anandamide acts as an intracellular messenger amplifying Ca2+ influx via TRPV1 channels. *EMBO J.* 24 3026–3037 16107881 10.1038/sj.emboj.7600784 PMC1201361

[B85] Wan Y. Chong L.W. Evans R. M. (2007). PPAR-gamma regulates osteoclastogenesis in mice. *Nat.Med.* 13 1496–1503 18059282 10.1038/nm1672

[B86] Whyte L. S. Ford L. Ridge S. A. Cameron G. A. Rogers M. J. Ross R. A. (2012). Cannabinoids, and bone: endocannabinoids modulate human osteoclast function in vitro. *Br. J. Pharmacol.* 165 2584–2597.. 21649637 10.1111/j.1476-5381.2011.01519.x PMC3423262

[B87] Whyte L. S. Ryberg E. Sims N. A. Ridge S. A. Mackie K. Greasley P. J. (2009). The putative cannabinoid receptor GPR55 affects osteoclast function in vitro and bone mass in vivo. *Proc. Natl. Acad. Sci. U.S.A.* 106 16511–16516 19805329 10.1073/pnas.0902743106 PMC2737440

[B88] Wu R. W. Lin T. P. Ko J. Y. Yeh D. W. Chen M. W. Ke H. C. (2011). Cannabinoid receptor 1 regulates ERK and GSK-3beta-dependent glucocorticoid inhibition of osteoblast differentiation in murine MC3T3-E1 cells. *Bone* 49 1255–1263 21914493 10.1016/j.bone.2011.08.022

